# Supplementary material for: Foliar Application of Chelated Sugar Alcohol Calcium Improves Photosynthesis and Tuber Quality under Drought Stress in Potatoes (Solanum tuberosum L.)
Source: Int J Mol Sci. 2023 Jul 30;24(15):12216. doi: 10.3390/ijms241512216 (PMC10418820; doi:10.3390/ijms241512216)
Supplement: Supplementary file 1 [file ijms-24-12216-s001.zip › Figure S1-2.pdf]

Supplementary Figures

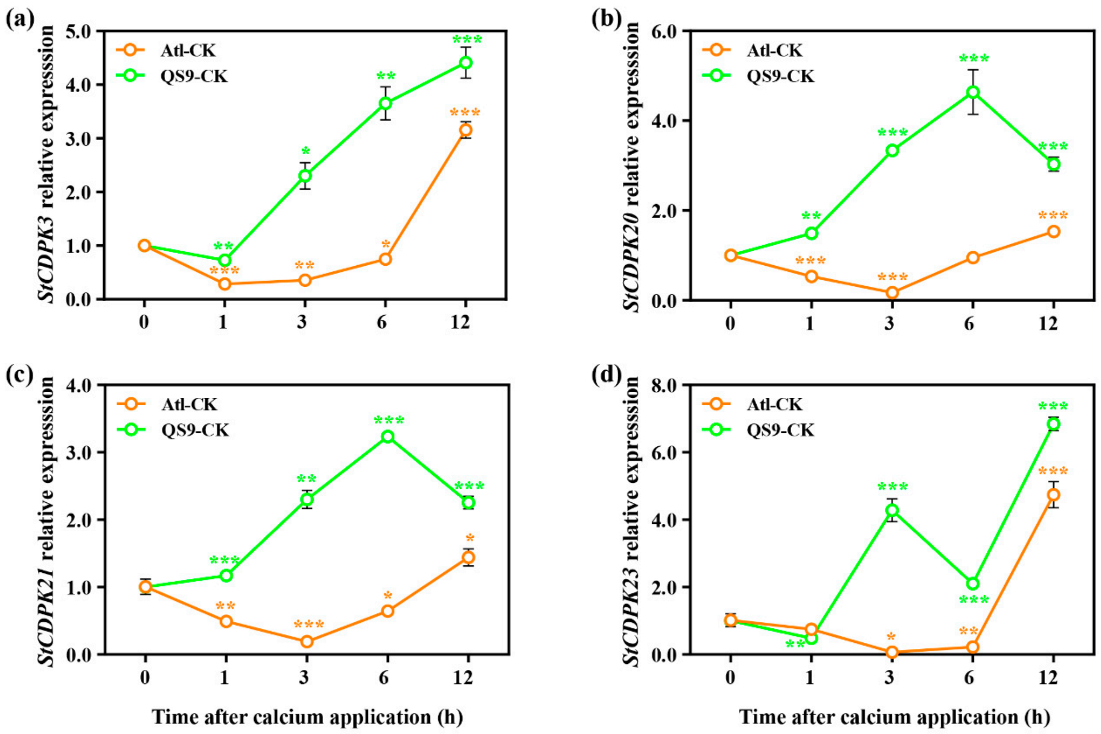

Figure S1. Effect of foliar application of Ca^{2+} on (**a**) *StCDPK3* (XM_006366477.2); (**b**) *StCDPK20* (NM_001318643.1); (**c**) *StCDPK21* (XM_006351851.2) and (**d**) *StCDPK23* (XM_015310592.1) of two potato cultivars ('Atlantic' and 'Qingshu 9') under normal conditions. 0 h is the control, a sample of untreated plants under normal conditions. Vertical bars indicate means ± s.d. The expression level was assessed using the $2^{-\Delta\Delta Ct}$ method, with *Actin* (XM_015308091.1) as an internal reference gene. Student's *t*-test was performed to evaluate the significance between different time points and 0 h. *, $p < 0.05$; **, $p < 0.01$; and ***, $p < 0.001$.

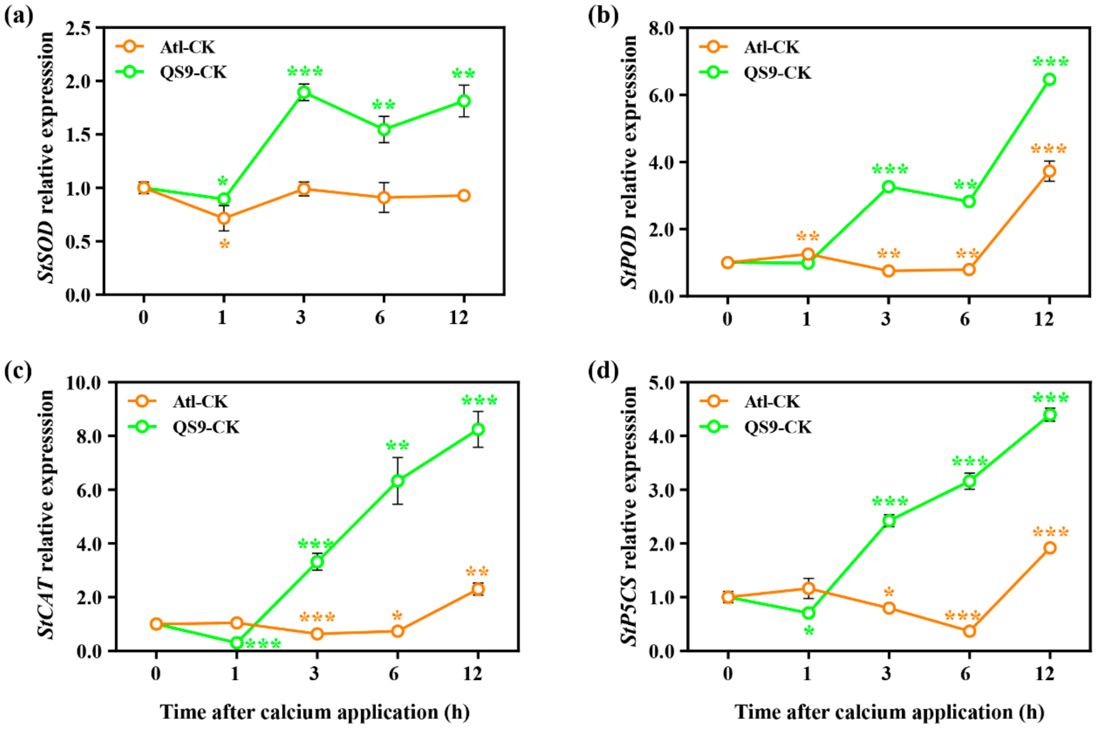

Figure S2. Effect of foliar application of Ca^{2+} on marker genes of physiological indicators (**a**) *StSOD* (XM_006358693.2); (**b**) *StPOD* (XM_006350817.2); (**c**) *StCAT* (NM_001287934.1) and (**d**) *StP5CS* (XM_006355200.2) of two potato cultivars ('Atlantic' and 'Qingshu 9') under normal condition. 0 h is the control, a sample of untreated plants under normal conditions. Vertical bars indicate means ± s.d. The expression level was assessed using the $2^{-\Delta\Delta Ct}$ method, with *Actin* (XM_015308091.1) as an internal reference gene. Student's *t*-test was performed to evaluate the significance between different time points and 0 h. *, $p < 0.05$; **, $p < 0.01$; and ***, $p < 0.001$.
